# Associations between Governmental Policies to Improve the Nutritional Quality of Supermarket Purchases and Individual, Retailer, and Community Health Outcomes: An Integrative Review

**DOI:** 10.3390/ijerph17207493

**Published:** 2020-10-15

**Authors:** Alyssa J. Moran, Yuxuan Gu, Sasha Clynes, Attia Goheer, Christina A. Roberto, Anne Palmer

**Affiliations:** 1 Department of Health Policy and Management, Johns Hopkins Bloomberg School of Public Health, Baltimore, MD 21205, USA; agoheer1@jhu.edu; 2 Department of International Health, Johns Hopkins Bloomberg School of Public Health, Baltimore, MD 21205, USA; yuxuangreen@gmail.com (Y.G.); sclynes1@jhmi.edu (S.C.); 3 Department of Medical Ethics and Health Policy, Perelman School of Medicine at the University of Pennsylvania, Philadelphia, PA 19104, USA; croberto@pennmedicine.upenn.edu; 4 Department of Health Behavior and Society, Johns Hopkins Bloomberg School of Public Health, Baltimore, MD 21205, USA; apalmer6@jhu.edu; 5 Center for a Livable Future, Johns Hopkins Bloomberg School of Public Health, Baltimore, MD 21202, USA

**Keywords:** food purchase, policy, retail food environment, food and beverage, grocery, federal nutrition assistance programs, beverage tax, menu labeling, financial incentives, health disparities

## Abstract

Supermarkets are natural and important settings for implementing environmental interventions to improve healthy eating, and governmental policies could help improve the nutritional quality of purchases in this setting. This review aimed to: (1) identify governmental policies in the United States (U.S.), including regulatory and legislative actions of federal, tribal, state, and local governments, designed to promote healthy choices in supermarkets; and (2) synthesize evidence of these policies’ effects on retailers, consumers, and community health. We searched five policy databases and developed a list of seven policy actions that meet our inclusion criteria: calorie labeling of prepared foods in supermarkets; increasing U.S. Department of Agriculture (USDA) Supplemental Nutrition Assistance Program (SNAP) benefits; financial incentives for the purchase of fruit and vegetables; sweetened beverage taxes; revisions to the USDA Special Supplemental Nutrition Program for Women, Infants, and Children (WIC) food package; financial assistance for supermarkets to open in underserved areas; and allowing online purchases with SNAP. We searched PubMed, Econlit, PsycINFO, Web of Science, and Business Source Ultimate to identify peer-reviewed, academic, English-language literature published at any time until January 2020; 147 studies were included in the review. Sweetened beverage taxes, revisions to the WIC food package, and financial incentives for fruits and vegetables were associated with improvements in dietary behaviors (food purchases and/or consumption). Providing financial incentives to supermarkets to open in underserved areas and increases in SNAP benefits were not associated with changes in food purchasing or diet quality but may improve food security. More research is needed to understand the effects of calorie labeling in supermarkets and online SNAP purchasing.

## 1. Introduction

Poor diet is widely considered a public health crisis, contributing to many of the leading causes of morbidity and mortality in the United States (U.S.) and globally [1,2]. There is growing recognition that dietary behaviors are shaped by the environments in which people live, learn, work, and play, and public health interventions increasingly target these settings [3]. Compared to nutrition interventions aimed at individuals or groups, upstream interventions designed to alter the environments in which people make food and beverage choices may be more effective for improving health, and are less costly to implement in the long-term [4,5]. For example, environmental interventions to treat obesity, such as sugary drink taxes and reductions in child-directed television advertising, are shown to be more cost-effective than commonly reimbursed clinical interventions, such as nutrition counseling or bariatric surgery [5].

In the U.S., supermarkets are natural and important settings for implementing environmental interventions to improve healthy eating. These stores, which generate more than $2 million annually in sales volume, are the primary retail store choice for the vast majority of U.S. households [6]. According to data from the U.S. Department of Agriculture (USDA) Food Acquisition and Purchase Survey, in 2012–2013, 89% of households did their primary shopping at supermarkets or other large grocers, with only 5% doing their primary shopping at other stores (e.g., convenience or dollar stores) [7]. During this time, supermarket purchases made up the majority of calories purchased by U.S. households (65%) and accounted for between 56% (households participating in the USDA Supplemental Nutrition Assistance Program [SNAP]) and 64% (higher income non-participating households) of household food expenditures [6]. Restaurant closures necessitated by the COVID-19 pandemic have likely increased reliance on supermarkets as a primary food source for many households.

The in-store environment is a well-recognized and powerful driver of dietary behaviors in supermarkets. Prior work has documented the important role of in-store food and beverage marketing, including availability, affordability, prominence, and promotion, in shaping consumer choices [8]. While these strategies hold promise for promoting healthy choices, they are often used to increase purchases of ultra-processed, nutritionally-poor products. A study of nearly 70 food retailers in three states found that sugary drinks were the most commonly promoted beverage, displayed in an average of 25 locations throughout the store [9]. National survey data show that the nutritional quality of purchases from supermarkets is generally poor, with diet quality scores (measured using the Healthy Eating Index 2010) closely mirroring those from national surveys of dietary intake [6,10].

Prior research has assessed the effectiveness of in-store promotions for healthy foods, finding that changes to product pricing, availability, packaging, display, signage, and labels are associated with consumer purchasing in the short-term [8]. Implementing these interventions long-term and scaling them across the nation’s more than 30,000 supermarkets, however, has proven challenging [11]. Grocery stores operate at low margins and rely on trade fees and discounts from food and beverage companies for revenue [12]. These fees often favor the largest manufacturers and distributors, allowing them to control which products are stocked and how items are promoted in the store. It is estimated that supermarkets collect more than $50 billion a year in trade fees and discounts, with fees accounting for a large proportion of total grocery revenue relative to sales (although fees vary greatly by product, manufacturer, and store type) [11,12]. These exorbitant financial incentives make voluntary interventions to promote healthy purchases difficult to implement in supermarkets without food and beverage company buy-in.

In the absence of widespread and sustained voluntary action, governmental policies could help increase healthy purchases and decrease unhealthy purchases in the supermarket setting. To this end, several policies have been implemented across the U.S., and many studies have been conducted to evaluate their effects. This integrative review aims to synthesize the academic literature on this topic by: (1) identifying U.S. governmental policies, including regulatory and legislative actions of federal, tribal, state, and local governments, designed to promote healthy choices in supermarkets; and (2) summarizing the available evidence related to these policies’ effects on retailers, consumers, and community health via changes to the supermarket environment. The objective of this review is to provide researchers and policymakers with information on existing policy options, their relative effectiveness in improving dietary behaviors, co-benefits or unintended consequences (e.g., impacts on retailer revenues or community economic development), and areas in need of further research. Although previous reviews have examined the effects of some of these policies, in isolation, on individual dietary or health outcomes, this will be the first, to our knowledge, to compare a wide range of outcomes across multiple policy approaches.

## 2. Methods

**Figure 1:**
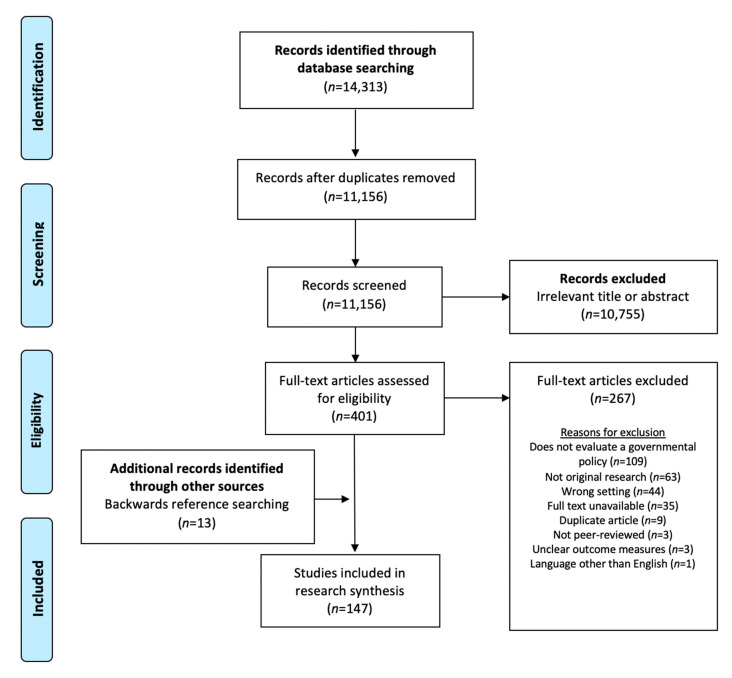
Preferred Reporting Items for Systematic Reviews and Meta-Analyses (PRISMA) Flow Diagram.

This review sought to answer two research questions: (1) which governmental policies in the U.S. aim to promote healthy choices in supermarkets; and (2) what is known about the effects of these policies on retailers, consumers, and communities? To answer these questions, we conducted searches of the peer-reviewed, academic, English-language literature published until January 2020. We searched PubMed, Econlit, PsycINFO, Web of Science, and Business Source Ultimate to identify papers spanning the economics, public health, marketing, consumer behavior, and business literature. Methods used to select and analyze results were consistent with the Preferred Reporting Items for Systematic Reviews and Meta-Analyses (PRISMA) guidelines (Figure 1) [13].

### 2.1. Policy Identification

To answer the first research question, we reviewed five nutrition policy databases: (1) World Cancer Research Fund International’s NOURISHING Database [14]; (2) The World Health Organization Global Database on the Implementation of Nutrition Action [15]; (3) Healthy Food Access Portal [16]; (4) Growing Food Connections Local Government Policy Database [17]; and (5) Healthy Food Policy Project [18]. From each database, two authors (Y.G. and S.C.) recorded the name, brief summary, date of enactment, and locale of government regulatory or legislative actions, meeting the following inclusion criteria: (1) implemented in a supermarket setting; (2) enacted in the U.S. as of 26 September 2019; and (3) intended to promote healthy food purchases (with policy intent inferred by the authors based on subject matter expertise). Policies that had been proposed but not enacted; policies that may influence food or beverage choices without changes to retailer practices (e.g., food formulation or front-of-package labeling policies); policies affecting only small stores or other non-supermarket settings (e.g., a healthy staple food ordinance); or policies that may affect food and beverage purchases, but were not designed with such intent (e.g., small sales taxes on soda or federal mandates requiring states to issue Supplemental Nutrition Assistance Program [SNAP] benefits no more than once monthly) were not included.

### 2.2. Search Strategy and Inclusion Criteria

Policies identified in this initial search were used to define our search terms, which were developed in collaboration with an informationist with expertise in public health policy (Appendix A). Papers were included if they met all of the following criteria: (1) peer-reviewed, original research; (2) conducted in the U.S.; (3) written in English; (4) evaluated a governmental policy, as defined above; and (5) assessed outcomes related to retailers (e.g., supermarket or manufacturer sales, revenue, or employment), consumers (e.g., dietary intake, food purchases, food security, body mass index), or communities (e.g., healthcare costs). In addition to quantitative evaluations, implementation, mixed methods, and qualitative research were included. Studies were excluded if they: (1) only assessed outcomes in small stores or other non-supermarket settings (e.g., availability of healthful foods in convenience stores); (2) described policy development but did not evaluate policy effects; or (3) described public comments or public opinion prior to policy enactment. After each search, duplicates were removed and titles, abstracts, and full texts were independently screened for inclusion by two authors (Y.G. and S.C.) using Covidence, a software for evidence synthesis (Covidence, Melbourne, Australia). Backward reference searching of included articles and reviews was conducted to identify additional papers. A third author (A.J.M.) was available to resolve disagreements (Figure 1).

### 2.3. Evidence Synthesis

Research was catalogued in alignment with the NOURISHING framework, which classifies policies into specific actions (e.g., posting calories on menu boards) within ten broad approaches (e.g., nutrition labeling). Three authors (Y.G., S.C., and A.J.M.) read each article and abstracted data on study setting (U.S. census region and urbanicity); design (controlled experimental, controlled quasi-experimental, descriptive (quantitative, including uncontrolled interventions, microsimulations, and modeling studies); descriptive (qualitative), or mixed/multiple methods); and population (adults, children, households, or other (e.g., stores, prices, benefit redemptions)). For each study, one study design was selected, but multiple settings and populations could be selected. Given the large and varied amount of research reviewed, additional quantitative or meta-analysis was not possible. Instead, a summary of findings, including outcomes, approaches, and research gaps, was generated using thematic analysis and narrative synthesis. Results are presented by policy action and approach.

## 3. Results

We identified 147 peer-reviewed academic research studies for inclusion in this review. These studies evaluated seven policy actions within three policy approaches (Table 1). The majority of studies used a descriptive, quantitative (52%) or controlled, quasi-experimental design (37%); were conducted among adults (48%); and used national data (44%) or were set in the Northeast (30%) (Table 2). Few studies used experimental (7%), qualitative (4%), or mixed/multiple methods designs (3%); were conducted among children (24%); or were set in the south (6%), Midwest (7%), or rural areas (6%).

### 3.1. Nutrition Labeling

#### Require Calorie Labeling of Prepared Food in Supermarkets

Few (*n* = 3) studies examined outcomes related to calorie labeling of prepared foods in supermarkets [35,36,37] and only one estimated the effects of calorie labels on food choices in a real-world supermarket setting [35]. Bachman et al. studied 393 women before and after calorie labeling in nine locations of a regional supermarket using a quasi-experimental design. Only 16% of study participants exposed to calorie labeling reported noticing the labels, and calorie labels did not influence food choices, although the sample size was small. In two studies, people trying or wanting to lose weight were more likely to rate calorie labels as important than people who were satisfied with their current weight [35,37]. Both studies used self-reported measures of consumer perceptions to assess the impact of calorie labeling; no studies have measured outcomes using validated dietary assessment surveys or objective food purchase data.

### 3.2. Economic Tools to Address Food Affordability and Purchase Incentives

#### 3.2.1. Increase Supplemental Nutrition Assistance Program (SNAP) Benefits

Thirteen studies assessed the effect of increased SNAP benefits (see Table 1 for description of policy) on household expenditures [38,39,40,41], food security [24,40,42,43], dietary behaviors [24,40,43,44,45,46,47], obesity [25], and healthcare utilization [48], with the majority using experimental (*n* = 2) or quasi-experimental (*n* = 8) designs. Studies indicate that increasing SNAP during the American Recovery and Reinvestment Act (ARRA) increased food-at-home expenditures but not food-away-from home expenditures [39,41,47], increased the share of benefits spent at superstores versus small stores [38], and increased spending on other necessary goods and services, including housing (mortgage, rent, utilities), transportation, and educational tuition [41]. Studies consistently demonstrated improvements in food security resulting from the ARRA benefit increase and Summer Electronic Benefits Transfer (EBT), as well as decreases in food security when the ARRA benefit increase ended [24,40,42,43]. Two studies found that benefit increases resulting from ARRA significantly reduced, but did not eliminate, declines in energy intake at the end of the benefit month [44,45], and one study found that ARRA was associated with a 65% reduction in outstanding medication needs due to cost among SNAP-eligible children [48].

The evidence for improving dietary behaviors and obesity is mixed. Most studies have found null or limited effects of a SNAP benefit increase on adult dietary quality [43,44,46,47]. A microsimulation study that directly compared the effects of an increase in SNAP benefits with a targeted subsidy on fruits, vegetables, and milk found that for the cost, targeted subsidies were more than ten times as effective in reducing deficiencies of recommended food groups [47]. One study of Summer EBT observed a small increase in children’s fruit and vegetable, whole grain, and dairy intake, but no change in consumption of unhealthful foods and beverages [24,40]. One study showed a reduction in BMI among adults; however, that study assessed the impact of an indirect increase in SNAP benefits resulting from children’s enrollment in school (and thus, participation in school meal programs), and may have been confounded by other changes affecting weight that correspond with school enrollment, such as changes in childcare expenses [25].

#### 3.2.2. Provide Financial Incentives for Fruits and Vegetables to Low-Income Households

Nineteen studies examined the impact of supermarket fruit and vegetable subsidies, incentives, vouchers, or prescriptions targeted towards low-income households or individuals [47,49,50,51,52,53,54,55,56,57,58,59,60,61,62,63,64,65,66]. Results from randomized trials and natural experiments consistently demonstrate increases in household fruit and vegetable purchases or adult fruit and vegetable intake when incentives are targeted towards SNAP participants [50,51,52,53,54,55,56,57,58]; yet, few studies have been conducted with children [52]. Studies assessing substitution found little evidence that fruit and vegetable incentives changed unhealthful food intake or expenditures [51,52,53,59]. Intervention effects, however, may not be sustained after the financial incentive ends [54,57]. Although incentive programs in supermarkets would have high start-up costs if implemented nationally, they are expected to be cost-saving in the long-term, largely due to reductions in type 2 diabetes, heart disease, and stroke [60,61,62,63]. Compared to research on SNAP incentives, there are limited data on produce prescription programs, which, to date, have been most frequently implemented in farmers’ markets or other limited-service food retail settings [49].

When the design and delivery of incentive programs are considered, the impacts on purchasing and consumption may increase with the size of the incentive. For example, the Healthy Incentives Pilot, which provided a 30% incentive on fruits and vegetables, saw a 26% increase (equivalent to approximately ¼ serving per day) in consumption among adults participating in SNAP, while the Shop Five for ME study, which provided a 50% incentive, found a 54% increase in fruit and vegetable purchases among SNAP households [52,53]. Additionally, incentives delivered as same-day discounts versus future rebates, administered electronically versus as paper coupons or vouchers, and offered without a minimum purchase requirement may increase uptake by lower-income households [52,55,56,57]. Frequent engagement with participating households and store staff about how the incentives work, which items qualify, and where they can be used may also be important for increasing utilization [49,52,56,58]. Complementary interventions focused on changing policies or environments to reduce unhealthy food purchases appear more effective in improving total diet quality at the population level than nutrition or cooking education, which tend to have low participation [51,52,59,61,63].

#### 3.2.3. Tax Sweetened Beverages

Forty-eight studies evaluated the impact of sweetened beverage excise taxes on a variety of behavioral, economic, and health outcomes in the U.S [5,60,63,64,65,67,68,69,70,71,72,73,74,75,76,77,78,79,80,81,82,83,84,85,86,87,88,89,90,91,92,93,94,95,96,97,98,99,100,101,102,103,104,105,106,107,108,109]. Evidence from real-world natural experiments shows that these taxes increase retail prices, although pass-through (the proportion of the tax that is passed on to the consumer) varies by city, store type, and beverage type and size [70,71,73,77,98,100]. Excise taxes reduce sales of taxed beverages, but the magnitude of the reduction is highly variable across cities [68,98,100,101]. For example, a $0.01 per ounce tax on calorically sweetened beverages in Berkeley, California was associated with a 9.6% decline in sugary drink sales volume in Berkeley supermarkets after one year [100]. In a natural experiment, a $0.015 per ounce tax on calorically and non-calorically sweetened beverages in Philadelphia, Pennsylvania was associated with a 38% reduction in taxed beverage volume sales after accounting for people who avoided the tax by purchasing sweetened beverages outside city limits [98]. Differences across studies may be due to baseline purchasing habits or income of the population, size of the tax, types of beverages included in the tax, the proportion of the population able to easily avoid the tax (i.e., by shopping in a bordering city), or tax salience.

There are less consistent data on changes in beverage consumption, total diet, or health outcomes, particularly among children. Evidence on dietary intake is mixed, possibly due to measurement error in dietary assessment tools and inadequate sample sizes [69,78,90,100,108]. For example, one study found a statistically significant reduction in sales of sugary drinks and increase in sales of untaxed beverages, but no statistically significant change in adult beverage intake one year after the Berkeley tax [100]. Another study one year after a Philadelphia tax found no changes in children’s beverage intake overall, but significant reductions in sugary drink intake among children who were high consumers prior to the tax [71]. There are limited quantitative data on the long-term (>1 year) effects of sweetened beverage taxes; one study of the earliest tax in Berkeley found an average reduction in sugary drink consumption (−0.55 times/day) and increased water consumption (+1.02 times/day) among adults 3 years after the tax [90]. Modeling studies with varying assumptions consistently show taxes improve long-term health outcomes related to obesity, cardiovascular disease, and diabetes among adults, and reduce childhood obesity [5,60,63,64,65,84,87,91,93,94,96,99,103,104]. Several of these studies suggest a tax on calories or sugar in beverages may better target health harms and encourage industry reformulation, but these taxation strategies have not yet been implemented in the U.S [85,86,106,109].

Economic research shows taxes are highly cost-saving from a public health and societal perspective, but may be costly to industry [104], with some studies documenting reduced supermarket combined sales [98], reduced sugar producer revenues [76,104], and increased cross-border shopping in cities with a tax (which may not be detrimental to supermarkets if shoppers visit the same chain in a different city) [69,70,98,100]. Although the food and beverage industries frequently voice concerns over job loss resulting from taxation, no job loss in these industries within the first year of a tax has been documented [89,97]. Strong and consistent evidence shows that beverage taxes raise revenue for city programs, such as parks and early childhood education, and these investments may affect social determinants of health [76,88]. For example, a simulation of Philadelphia’s tax found that investments in quality pre-kindergarten would further reduce sugary beverage consumption among young children by 8% [88].

#### 3.2.4. Revise Composition and Quantities of Foods Provided through the USDA Special Supplemental Nutrition Program for Women, Infants and Children (WIC)

Forty-four studies assessed the association between the 2009 WIC food package revisions and availability of foods and beverages in supermarkets; purchases, redemptions, or dietary intake among WIC participants; obesity in early childhood; perinatal and birth outcomes; or outcomes related to breastfeeding [110,111,112,113,114,115,116,117,118,119,120,121,122,123,124,125,126,127,128,129,130,131,132,133,134,135,136,137,138,139,140,141,142,143,144,145,146,147,148,149,150,151,152,153]. There is consistent evidence of an association between the WIC food package revisions and improvements in household food purchases and dietary intake among both adults and children [111,114,115,116,119,121,135,136,137,138,139,140,145,147,148,149,151]. Specifically, revisions to the food package are associated with improvements in total diet quality, increases in fruit, vegetables, whole grains, dietary fiber, and low-fat dairy, and reductions in full-fat dairy, saturated fat, and juice (with no evidence of complete substitution to other sugary drinks). The cash-value voucher, in particular, increased the perceived value of the program for many participants, although voucher redemption varied across communities and may be limited in some areas by poor access to fresh fruits and vegetables or negative store experiences [112,113,117,123,128,131,132,146]. Impacts of the revisions on breastfeeding are mixed, with some studies showing increases in breastfeeding initiation [129,153], others showing no effect [118], and none finding a relationship with breastfeeding at six months [129,153]. Recent research using interrupted time series or controlled quasi-experimental designs show improvements in maternal and child health outcomes resulting from the food package changes, including reductions in infant and young child obesity [125,126,127,130], improvements in infant birth weight outcomes (low birth weight, small for gestational age, and large for gestational age) [120], and reductions in maternal weight gain and preeclampsia [120].

With regard to the retail food environment, several studies have documented changes in WIC food availability, variety, quality, or pricing after implementation of the food package revisions and minimum stocking requirements [133,134,141,142,143,144,152]. While outcomes have generally been positive for small and medium-sized stores (i.e., greater availability and lower prices of healthful foods), results in supermarkets and mass merchandisers are mixed, likely due to the wide variety of food options offered in these stores at baseline. It is important to note that, in addition to the federal requirements, states have the authority to establish stronger stocking requirements for authorized retailers and there is substantial variation in regulatory guidance across states [142]. Variation in minimum stocking standards, as well as other flexibilities in how WIC programs are administered at the state and local levels, may partially explain observed differences in program impacts across localities; however, this has not been well studied [130].

### 3.3. Incentives and Rules to Create a Healthy Retail Environment

#### 3.3.1. Provide Financial Assistance to Supermarkets to Locate in Underserved Areas

Twenty-two studies assessed the impacts of new supermarkets locating in underserved areas. Though many community residents support the introduction of a new supermarket [154], studies, including many using controlled, quasi-experimental designs, show low adoption of the new supermarket [155,156,157] and little or no improvement in body mass indices [155,158,159,160,161,162], household food purchases [156,157,163,164], or dietary intake [155,156,157,158,159,160,165] attributable to the new store. Similarly, modeling studies show these interventions are less cost-effective for supporting the introduction of new stores and increasing shopping at supermarkets than policies that increase SNAP benefits or coverage [38,166]. However, distance to the store, health and economic characteristics of the community, and baseline shopping habits within the population, may be important effect modifiers [167,168]. Additionally, supermarkets may positively impact health independent of effects on diet. One longitudinal natural experiment showed no improvement in dietary intake, but reductions in SNAP participation, food insecurity, and diagnoses of high cholesterol and arthritis one year after the opening of a new store [169]; however, the mechanism through which these positive health effects occurred is unclear. Several studies have suggested that investments in healthy retail may positively impact health by improving economic opportunity, social cohesion, or safety, but these mechanisms have not been studied [168,170,171].

#### 3.3.2. Allow Payment for Online Grocery Purchases with SNAP

No research has studied the effects of online grocery shopping on the diets of SNAP participants, but several recent studies provide insight on the availability and uptake of online SNAP purchases [172,173,174,175]. Three studies have shown that, while online grocery can address transportation barriers and food availability, perceptions related to higher food costs online, lack of control over food quality, and distrust of the online shopping process may prevent SNAP participants from utilizing these services [173,174,175]. One attempted trial was unable to recruit enough SNAP participants to make online grocery purchases, mainly due to participants’ perceived lack of control over the quality of food selected [174]. Additionally, a recent study found that online grocery delivery services disproportionately serve urban areas; services are rarely available in rural areas [172]. It will be important to continue to monitor equitable access to online grocery shopping and delivery over the course of the COVID-19 pandemic, particularly in communities and sub-populations at the highest risk of infection.

## 4. Discussion

This integrative review aimed to identify governmental policies enacted in the U.S. to promote healthy choices in supermarkets and to synthesize the academic literature on these policies’ effects. We identified 147 papers in seven policy areas: calorie labeling, SNAP benefit increases, financial incentives to purchase fruit and vegetables, sweetened beverage taxes, revisions to the WIC food package, financial assistance for supermarkets to locate in underserved areas, and allowing online purchases with SNAP. The majority of identified papers were related to sweetened beverage taxes (33%), followed by revisions to the WIC food package (30%), and financial incentives for supermarkets (15%); few studies assessed calorie labeling of prepared foods in supermarkets (2%) or online SNAP (3%). Most studies leveraged natural experiments to evaluate policy effects, utilizing controlled, quasi-experimental study designs, microsimulation or agent-based modeling, longitudinal approaches, and interrupted time series methods; very few studies employed experimental, qualitative, or mixed methods approaches. With regard to population, many studies were conducted among adults, except in the case of WIC food package revisions, in which studies of young children were more common. Studies frequently used national data or data collected in Northeastern or Western U.S. cities.; far fewer studies were set in the Southern or Midwestern U.S. or in rural areas.

When effects were compared across policy action, we found consistent evidence, including from real-world randomized trials and natural experiments, of an association between economic tools to address food affordability and dietary behaviors. Specifically, sweetened beverage taxes were associated with increased prices and decreased purchases of taxed beverages, revisions to the WIC food package were associated with improvements in total diet quality and maternal/child health outcomes, and fruit and vegetable incentives increased purchases and consumption of discounted foods. In modeling studies, all three policies reduced the incidence of cardiometabolic diseases and were cost-effective in the long-term, but those restricting or discouraging consumption of unhealthful foods (i.e., taxes, WIC revisions) showed greater gains than those solely encouraging consumption of healthful foods (i.e., incentives). When incentives were paired with restrictions or taxes on unhealthful purchases, however, their combined effects on dietary behaviors were greater than those of any single policy action. This highlights the importance of multiple, synergistic policy interventions delivered together.

In contrast to the economic levers described above, financial assistance for supermarkets to open in underserved neighborhoods and increases in the SNAP benefit amount had little effect on diet, but reduced food insecurity. Food insecurity is associated with a wide range of negative outcomes, including increased risk of obesity and cardiometabolic diseases [176], poor mental health [177], and poor early childhood development [178]. Thus, these policy interventions could improve long-term health by reducing food insecurity, but these mechanisms have not yet been studied. Longitudinal natural experiments are needed to understand the role of supermarkets in neighborhood revitalization and the complex relationship between economic development strategies and improved health of neighborhood residents.

This review exposed several gaps in the literature that could be addressed in future research. First, research on calorie labeling of prepared foods in supermarkets and online SNAP is nascent and could be examined using natural experiments or interrupted time series designs. Second, very little research has been conducted in rural areas or in the Southern or Midwestern U.S.—regions with a disproportionately high prevalence of obesity and related health conditions [179]. Similarly, few studies assessed policy impacts on racial or socioeconomic disparities. While some policies may not substantially improve average dietary intake, they may contribute to improving equity. Third, although this review sought to include a wide range of outcomes, most studies evaluated policy effects on food security, household purchases, dietary intake, or obesity. Other important health-related outcomes, such as changes to social norms, parental feeding practices, and modeling of healthful behaviors, are needed and could be assessed using qualitative or mixed methods. Fourth, very few studies examined outcomes of importance to retailers, such as customer loyalty or sales revenue, which could help foster retail partnerships and industry buy-in. Fifth, policy implementation was rarely addressed. Implementation of federal policies often varies at the state level, and state and local policies with similar goals often differ in scope. This variation in implementation could explain variation in outcomes across settings, but it has not been quantitatively or qualitatively assessed. Similarly, process outcomes, such as policy adoption, acceptability, or fidelity, were infrequently measured and could help explain null effects. Lastly, many quasi-experimental studies were limited by small sample sizes and crude dietary assessment tools, which may have limited investigators’ abilities to detect small policy effects. Investigators should carefully consider required sample sizes and appropriate dietary assessment methods to avoid false null findings, which can be detrimental to policy and advocacy efforts.

### Strengths and Limitations

This review has several limitations and strengths that should be considered. In line with the project aims, a large amount of literature was reviewed and, thus, strength and quality of evidence was not quantitatively assessed but rather qualitatively synthesized. As a means to limit included studies to the highest quality papers, non-academic, non-peer reviewed sources were not included. This decision likely led to exclusion of some important evidence, such as reports commissioned by the USDA or other agencies or organizations. It also excludes industry reports, which may be more likely to assess outcomes relevant to retailers. We did not include food formulation, front-of-pack, or back-of-pack labeling policies in this review, although such policies could theoretically influence the types of products stocked or how they are priced or promoted within the store. Strengths of the study include extraction of relevant policies from five policy databases, comprehensive search strings and database searches across the psychology, economics, business, marketing, policy, and public health literature, inclusion of a wide range of effects on individuals, retailers, and community health, and narrative comparison of effects across seven distinct policy actions.

## 5. Conclusions

Governmental policies, particularly sweetened beverage taxes, revisions to the WIC food package, and financial incentives for fruits and vegetables, are associated with improvements in dietary behaviors. Providing financial incentives to supermarkets to open in underserved areas and increases in SNAP benefits are not associated with changes in food purchasing or diet quality but may improve food security. More research is needed to understand the effects of calorie labeling in supermarkets and allowing online purchases with SNAP.

## Figures and Tables

**Table 1:** Description of policy actions included in the review.

Policy Approach	Policy Action	Policy Description
Nutrition Label Standards and Regulations on The Use of Claims and Implied Claims on Food	Require Calorie Labeling of Prepared Food in Supermarkets (*n* = 3)	The 2010 Affordable Care Act mandated restaurants and similar food retail establishments with 20 or more locations nationwide disclose the number of calories in prepared foods on menus, menu boards, or in close proximity to food items (“calorie labeling”) (P.L. 111-148, §4205) [19]. Food retailers are also required to display a statement about daily calorie intake (“2000 calories a day is used for general nutrition advice, but calorie needs vary”) and must alert consumers that nutrition information for standard menu items is available upon request. The federal calorie labeling law differed from previously implemented state and local policies in that it expanded covered food retail establishments to include not only restaurants, but also supermarkets and other venues selling prepared foods. The Food and Drug Administration (FDA) published their final menu labeling rule in December 2014, issued draft industry guidance in 2017, and the law took effect on 7 May 2018. The following year, the FDA launched a consumer-facing nutrition education campaign to encourage the use of calorie information when eating out [20].
Use Economic Tools to Address Food Affordability and Purchase Incentives	Increase SNAP Benefits (*n* = 13)	SNAP provides monthly food benefits to approximately 36 million low-income Americans [21]. The majority (83%) of SNAP benefits are spent in supermarkets, totaling about $46 billion annually [21,22]. SNAP benefits are calculated on the assumption that households will spend 30% of their incomes on food, with SNAP bridging the gap between that contribution and the cost of the Thrifty Food Plan (a very low cost healthy diet, as determined by the USDA) [23]. Congressional action can increase the monthly SNAP benefit amount, and Congress has approved benefit increases in response to economic downturns. For example, in response to the great recession, the American Recovery and Reinvestment Act increased SNAP benefits by an average of 13.6% (about $80/month for a family of four) from April 2009 until November 2013 (P.L.111-5, §101). Benefit increases could also be provided by expanding Summer EBT for Children (Summer EBT), which provides benefits to families of students eligible for free or reduced-price lunch during the summer months, when school is not in session. Additionally, several policies may increase SNAP benefits indirectly, by reducing the number of meals provided to school-aged children by low-income households each week [24]. For example, policies that increase school participation in the Community Eligibility Provision, which allows schools or districts serving a certain percentage of eligible students to provide all students with free breakfast and lunch, may indirectly increase SNAP benefits among households with children by increasing student participation in school meal programs [25].
	Provide Financial Incentives for Fruits and Vegetables to Low-Income Households (*n* = 19)	The 2014 Farm Bill provided $100 million in mandatory funding for the Food Insecurity and Nutrition Incentive grant program to support programs that provide SNAP participants with financial incentives for the purchase of fruits and vegetables (P.L. 113-79, §4208) [26]. The 2018 Farm Bill expanded and permanently reauthorized this program, renamed the Gus Schumacher Nutrition Incentive Program (GusNIP), and increased funding to $250 million over five years (P.L. 115-334, §4205). Through GusNIP, the USDA also authorized the Produce Prescription Program, which provides funding to organizations to partner with healthcare providers to provide financial incentives for fresh fruits and vegetables to low-income people at risk of diet-related health conditions.
	Tax Sweetened Beverages (*n* = 48)	In 2014, the city of Berkeley, California became the first U.S. city to pass a sweetened beverage excise tax of $0.01 per ounce [27]. Since then, a total of seven U.S. cities and the Navajo Nation have implemented similar taxes. All taxes except that of the Navajo Nation range from $0.01 to $0.02 per ounce. Sweetened beverage taxes typically apply to beverages with added sugar, but may also include drinks with low- or no-calorie sweeteners (e.g., the city of Philadelphia, Pennsylvania taxes both calorically and non-calorically sweetened beverages). Compared to a sales tax, which does not affect the posted retail price, excise taxes on beverage distributors can be passed on to consumers through retail price increases (“pass-through”).
	Revise the WIC Food Package (*n* = 44)	In October 2009, as required under the Child Nutrition Act (P.L. 111-296, §17), the USDA reviewed and revised the WIC food package to better align with the 2005 Dietary Guidelines for Americans, American Academy of Pediatrics Infant Feeding Guidelines, and 2006 Institute of Medicine recommendations [28,29,30]. The revisions included cash-value vouchers for fruits and vegetables, expanded whole grain and low-fat dairy options, reductions in whole milk, juice, eggs, and cheese, and additional incentives for breastfeeding [31]. WIC-authorized stores were required to stock a minimum variety of fruits, vegetables, and whole grain products. States have flexibility to determine which specific foods are included in the food package (within federal guidelines) and may require stricter minimum stocking standards for authorized stores.
Set Incentives and Rules to Create a Healthy Retail and Food Service Environment	Provide Financial Assistance to Supermarkets to Locate in Underserved Areas (*n* = 22)	The Healthy Food Financing Initiative (HFFI) was launched in 2011 and formally established at the USDA as part of the 2014 Farm Bill (P.L. 113-79, §4206) [32]. The goal of the program was to improve access to healthy foods in low-income communities by building supermarkets or farmers’ markets and improving the quality of foods offered in small stores through grants, loans, and tax incentives. Between 2011 and 2015, the HFFI awarded $195 million to community development organizations for nearly 1000 healthy food access projects in 35 states. Many municipalities operate similar programs at the state or local level [33].
	Allow Payment with SNAP for Online Grocery Purchases (*n* = 4)	The 2014 Farm Bill mandated the USDA Online Purchasing Pilot Program, which tests accepting SNAP/EBT for online grocery transactions (P.L. 113-79, §4011) [34]. In 2017, the USDA Food and Nutrition Service announced the selection of seven retailers in seven states to participate in the program, which was launched in Amazon, Shoprite, and Walmart in select zip codes in New York in April 2019. The program was meant to roll out among the remaining selected states and retailers over the next several years, but has rapidly expanded due to the COVID-19 pandemic. At the time of writing, forty states and five retailers were participating.

Note: The number of studies across all policy actions exceeds 147 because some studies addressed more than one policy action. Abbreviations: SNAP (Supplemental Nutrition Assistance Program); WIC (Supplemental Nutrition Program for Women, Infants, and Children); EBT (Electronic Benefits Transfer); HFFI (Healthy Food Financing Initiative); USDA (United States Department of Agriculture).

**Table 2:** Study design features of included articles, by policy action area.

Study Design Feature	Total (*n* = 147)	Calorie Labeling (*n* = 3)	SNAP Benefit Increase (*n* = 13)	Fruit and Vegetable Incentives (*n* = 19)	Sweetened Beverage Tax (*n* = 48)	WIC Food Package Revisions (*n* = 44)	Financial Assistance for Supermarkets (*n* = 22)	Online SNAP/EBT (*n* = 4)
**Study Design**								
Experimental	11 (7%)	0 (0%)	2 (15%)	9 (47%)	0 (0%)	0 (0%)	0 (0%)	0 (0%)
Quasi-experimental	55 (37%)	1 (33%)	8 (62%)	2 (11%)	17 (35%)	14 (32%)	13 (59%)	0 (0%)
Descriptive (Quantitative)	76 (52%)	2 (67%)	3 (23%)	7 (37%)	31 (65%)	25 (57%)	7 (32%)	1 (25%)
Descriptive (Qualitative)	6 (4%)	0 (0%)	0 (0%)	0 (0%)	0 (0%)	4 (9%)	1 (5%)	1 (25%)
Mixed or multiple methods	5 (3%)	0 (0%)	0 (0%)	1 (5%)	0 (0%)	1 (2%)	1 (5%)	2 (50%)
**Population**								
Adults	70 (48%)	2 (67%)	7 (54%)	13 (68%)	20 (42%)	12 (27%)	13 (59%)	3 (75%)
Children	35 (24%)	0 (0%)	3 (23%)	1 (5%)	10 (21%)	19 (43%)	2 (9%)	0 (0%)
Households	38 (26%)	0 (0%)	5 (38%)	9 (47%)	13 (27%)	8 (18%)	3 (14%)	0 (0%)
Other (e.g., stores)	33 (22%)	1 (33%)	1 (8%)	2 (11%)	13 (27%)	10 (23%)	5 (23%)	1 (25%)
**U.S. Census Region**								
National	64 (44%)	1 (33%)	11 (85%)	7 (37%)	26 (54%)	13 (30%)	5 (23%)	1 (25%)
Northeast	44 (30%)	1 (33%)	2 (15%)	8 (42%)	12 (25%)	9 (20%)	11 (50%)	1 (25%)
South	9 (6%)	0 (0%)	0 (0%)	0 (0%)	0 (0%)	5 (11%)	3 (14%)	1 (25%)
Midwest	10 (7%)	0 (0%)	1 (8%)	3 (16%)	2 (4%)	3 (7%)	1 (5%)	0 (0%)
West	30 (20%)	1 (33%)	1 (8%)	1 (5%)	10 (21%)	14 (32%)	2 (9%)	1 (25%)
Urban/Rural *								
Urban	54 (37%)	1 (33%)	1 (8%)	9 (47%)	18 (38%)	15 (34%)	11 (50%)	3 (75%)
Rural	9 (6%)	0 (0%)	0 (0%)	6 (32%)	0 (0%)	2 (5%)	1 (5%)	0 (0%)
Not specified or applicable	91 (62%)	2 (67%)	12 (92%)	8 (42%)	31 (65%)	27 (61%)	10 (45%)	1 (25%)

Note: The number of studies across all policy actions exceeds 147 because some studies addressed more than one policy action. Study design categories were mutually exclusive, but some studies addressed more than one geographic area and/or population. * As described in study (“cities” considered urban).

## Appendix A. Search Terms

**Search #1**
*Terms related to supermarket*
(grocer* OR supermarket OR store OR Retail* OR outlet OR e-commerce OR mercado)
AND
*Terms related to food and beverage*
food OR foods OR beverage* OR fruit OR fruits OR vegetable* OR snack* OR drink OR drinks OR lunch OR dinner* OR breakfast OR meat OR poultry OR beef OR chicken OR fish OR milk OR cheese OR yogurt OR juice OR soda OR grain OR grains OR meal OR bean OR beans OR nut OR nuts OR candy OR sweets OR cookies OR chips OR “ice cream” OR sugar OR salt OR sugar-sweetened OR “sugar sweetened” OR sweet OR nutrition* OR calorie OR calories
AND
*Terms related to policy*
policy OR policies OR Law OR laws OR regulat* OR ordinance* OR statute* OR tax OR taxes OR taxation OR incentive* OR “healthy food financing” OR subsid* OR “menu labeling” OR “calorie labeling” OR WIC OR “supplemental nutrition” OR “food stamps” OR access OR rule OR rules OR “retail expansion” OR “community development” OR “food trust” OR “food desert” OR loan OR loans OR “healthy food business” OR “healthy neighborhood” OR “federal nutrition program” OR “fresh food fund” OR “grocery financing” OR zoning OR “minimum stocking” OR “staple food” OR “excess food” OR “food waste” OR “grocery store development program” OR “closer to my grocer” OR license OR licensing OR permit OR permitting OR “Baton Rouge” OR frameworks OR “grocery access” OR “fresh food financing” OR “supermarket access” OR “healthy food center” OR “neighborhood development” OR “healthy families” OR “fresh food retailer”
**Search #2**
*Terms related to beverage taxes*
(“sweetened beverage*” OR “sugary drink*” OR “sugary beverage*”) AND (tax OR taxes OR taxation)
**Search #3**
*Terms related to SNAP benefit increases*
(ARRA OR (benefit AND increase)) AND “Supplemental Nutrition Assistance Program”
**Search #4**
*Terms related to WIC food package revisions*
((WIC AND (fruit* OR vegetable*) AND voucher)
OR
(((WIC OR “Special Supplemental Nutrition Program for Women, Infants, and Children”) AND (“food package OR revisions))) AND (“2014/04”[Date–Publication]: “3000”[Date-Publication]))
**Search #5**
*Terms related to the online purchasing pilot*
“online grocery” OR “online purchas* pilot” OR (online AND shopping AND “Supplemental Nutrition Assistance Program”)
**Search #6**
*Terms related to produce prescription programs*
((fruit OR vegetable* OR produce) AND (prescription OR rx)) AND (supermarket OR grocer*)
